# Supplementary material for: Longitudinal gut microbiome changes in immune checkpoint blockade-treated advanced melanoma
Source: Nat Med. 2024 Feb 16;30(3):785–96. doi: 10.1038/s41591-024-02803-3 (PMC10957474; doi:10.1038/s41591-024-02803-3)
Supplement: Supplementary file 1 — Supplementary Figs. 1–5 and methods. [file 41591_2024_2803_MOESM1_ESM.pdf]

nature medicine

Article

https://doi.org/10.1038/s41591-024-02803-3

Longitudinal gut microbiome changes in immune checkpoint blockade-treated advanced melanoma

In the format provided by the authors and unedited

Table of content

Supplementary figures **2**

Supplementary methods **11**

Supplementary figures

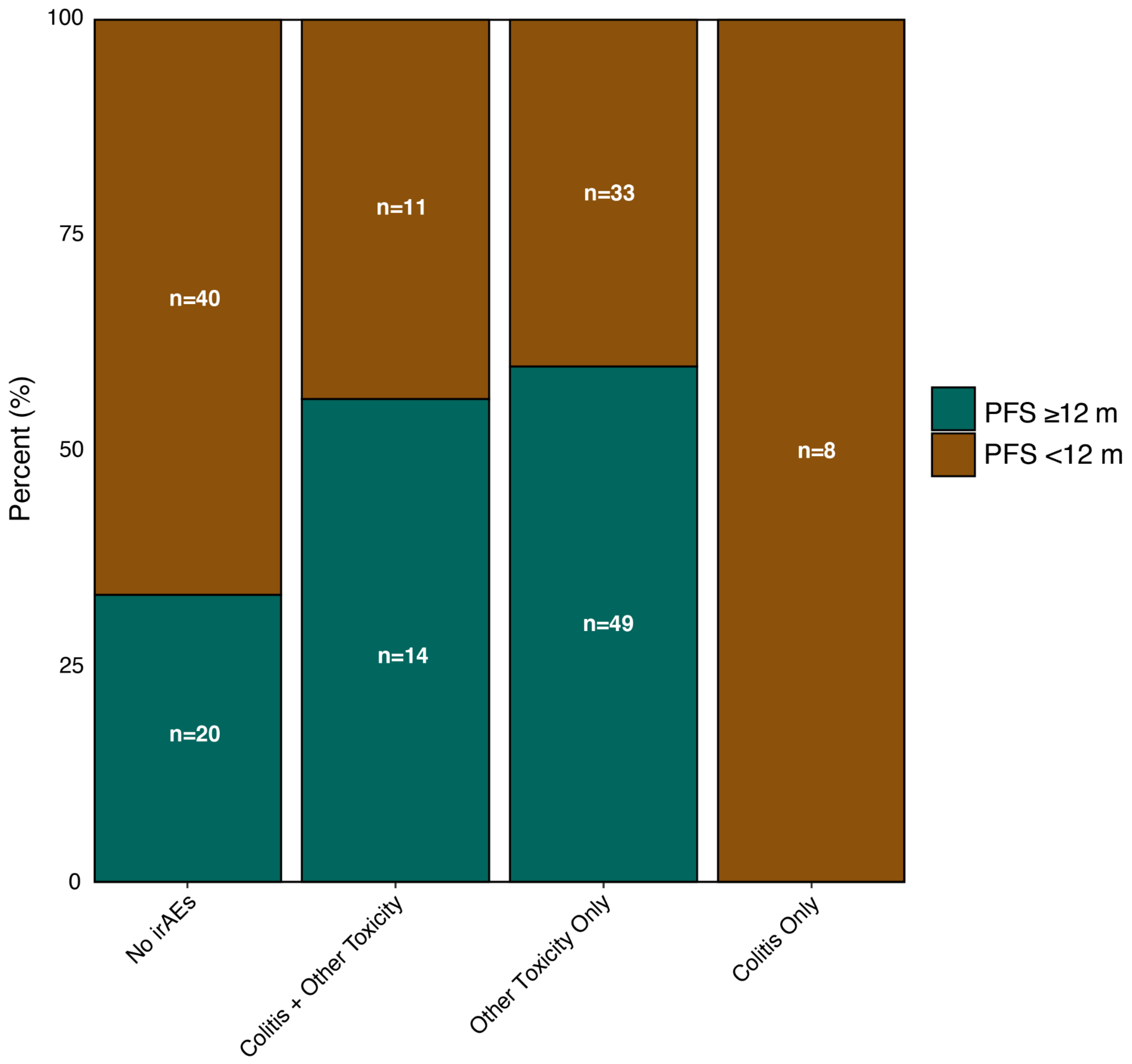

Figure S1. Relationship between PFS12 and irAEs. Mosaic plot for PFS12 and irAEs (separated into colitis and "other toxicity"). The mosaic plot shows how many patients with PFS≥12 and PFS<12 months did not develop any irAEs, how many developed both colitis and other toxicities, how many developed other toxicities but no colitis, and how many patients developed colitis only.

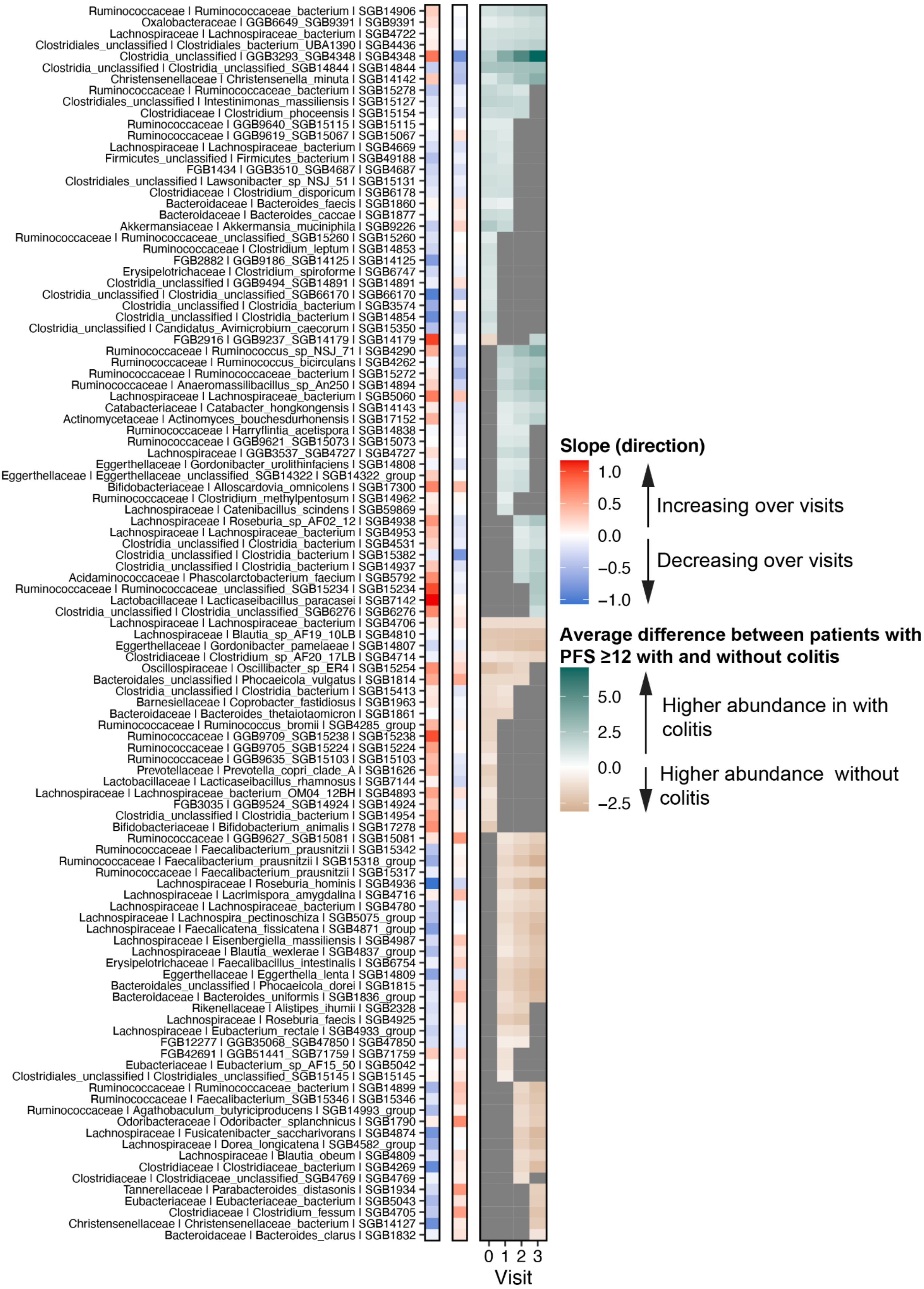
Ruminococcaceae | Ruminococcaceae_bacterium | SGB14906
Oxalobacteraceae | GGB6649_SGB9391 | SGB9391
Lachnospiraceae | Lachnospiraceae_bacterium | SGB4722
Clostridiales_unclassified | Clostridiales_bacterium_UBA1390 | SGB4436
Clostridia_unclassified | GGB3293_SGB4348 | SGB4348
Clostridia_unclassified | Clostridia_unclassified_SGB14844 | SGB14844
Christensenellaceae | Christensenella_minuta | SGB14142
Ruminococcaceae | Ruminococcaceae_bacterium | SGB15278
Clostridiales_unclassified | Intestinimonas_massiliensis | SGB15127
Clostridiaceae | Clostridium_phoceensis | SGB15154
Ruminococcaceae | GGB9640_SGB15115 | SGB15115
Ruminococcaceae | GGB9619_SGB15067 | SGB15067
Lachnospiraceae | Lachnospiraceae_bacterium | SGB4669
Firmicutes_unclassified | Firmicutes_bacterium | SGB49188
FGB1434 | GGB3510_SGB4687 | SGB4687
Clostridiales_unclassified | Lawsonibacter_sp_NSJ_51 | SGB15131
Clostridiaceae | Clostridium_disporicum | SGB6178
Bacteroidaceae | Bacteroides_faecis | SGB1860
Bacteroidaceae | Bacteroides_caccae | SGB1877
Akkermansiaceae | Akkermansia_muciniphila | SGB9226
Ruminococcaceae | Ruminococcaceae_unclassified_SGB15260 | SGB15260
Ruminococcaceae | Clostridium_leptum | SGB14853
FGB2882 | GGB9186_SGB14125 | SGB14125
Erysipelotrichaceae | Clostridium_spiroforme | SGB6747
Clostridia_unclassified | GGB9494_SGB14891 | SGB14891
Clostridia_unclassified | Clostridia_unclassified_SGB66170 | SGB66170
Clostridia_unclassified | Clostridia_bacterium | SGB3574
Clostridia_unclassified | Clostridia_bacterium | SGB14854
Clostridia_unclassified | Candidatus_Avimicrobium_caecorum | SGB15350
FGB2916 | GGB9237_SGB14179 | SGB14179
Ruminococcaceae | Ruminococcus_sp_NSJ_71 | SGB4290
Ruminococcaceae | Ruminococcus_bicirculans | SGB4262
Ruminococcaceae | Ruminococcaceae_bacterium | SGB15272
Ruminococcaceae | Anaeromassilibacillus_sp_An250 | SGB14894
Lachnospiraceae | Lachnospiraceae_bacterium | SGB5060
Catabacteriaceae | Catabacter_hongkongensis | SGB14143
Actinomycetaceae | Actinomyces_bouchesdurhonensis | SGB17152
Ruminococcaceae | Harryflintia_acetispora | SGB14838
Ruminococcaceae | GGB9621_SGB15073 | SGB15073
Lachnospiraceae | GGB3537_SGB4727 | SGB4727
Eggerthellaceae | Gordonibacter_urolithinfaciens | SGB14808
Eggerthellaceae | Eggerthellaceae_unclassified_SGB14322 | SGB14322_group
Bifidobacteriaceae | Alloscardovia_omnicolens | SGB17300
Ruminococcaceae | Clostridium_methylpentosum | SGB14962
Lachnospiraceae | Catenibacillus_scindens | SGB59869
Lachnospiraceae | Roseburia_sp_AF02_12 | SGB4938
Lachnospiraceae | Lachnospiraceae_bacterium | SGB4953
Clostridia_unclassified | Clostridia_bacterium | SGB4531
Clostridia_unclassified | Clostridia_bacterium | SGB15382
Clostridia_unclassified | Clostridia_bacterium | SGB14937
Acidaminococcaceae | Phascolarctobacterium_faecium | SGB5792
Ruminococcaceae | Ruminococcaceae_unclassified_SGB15234 | SGB15234
Lactobacillaceae | Lacticaseibacillus_paracasei | SGB7142
Clostridia_unclassified | Clostridia_unclassified_SGB6276 | SGB6276
Lachnospiraceae | Lachnospiraceae_bacterium | SGB4706
Lachnospiraceae | Blautia_sp_AF19_10LB | SGB4810
Eggerthellaceae | Gordonibacter_pamelaeae | SGB14807
Clostridiaceae | Clostridium_sp_AF20_17LB | SGB4714
Oscillospiraceae | Oscillibacter_sp_ER4 | SGB15254
Bacteroidales_unclassified | Phocaeicola_vulgatus | SGB1814
Clostridia_unclassified | Clostridia_bacterium | SGB15413
Barnesiellaceae | Coprobacter_fastidiosus | SGB1963
Bacteroidaceae | Bacteroides_thetaiotaomicron | SGB1861
Ruminococcaceae | Ruminococcus_bromii | SGB4285_group
Ruminococcaceae | GGB9709_SGB15238 | SGB15238
Ruminococcaceae | GGB9705_SGB15224 | SGB15224
Ruminococcaceae | GGB9635_SGB15103 | SGB15103
Prevotellaceae | Prevotella_copri_clade_A | SGB1626
Lactobacillaceae | Lacticaseibacillus_rhamnosus | SGB7144
Lachnospiraceae | Lachnospiraceae_bacterium_OM04_12BH | SGB4893
FGB3035 | GGB9524_SGB14924 | SGB14924
Clostridia_unclassified | Clostridia_bacterium | SGB14954
Bifidobacteriaceae | Bifidobacterium_animalis | SGB17278
Ruminococcaceae | GGB9627_SGB15081 | SGB15081
Ruminococcaceae | Faecalibacterium_prausnitzii | SGB15342
Ruminococcaceae | Faecalibacterium_prausnitzii | SGB15318_group
Ruminococcaceae | Faecalibacterium_prausnitzii | SGB15317
Lachnospiraceae | Roseburia_hominis | SGB4936
Lachnospiraceae | Lacrimispora_amygdalina | SGB4716
Lachnospiraceae | Lachnospiraceae_bacterium | SGB4780
Lachnospiraceae | Lachnospira_pectinoschiza | SGB5075_group
Lachnospiraceae | Faecalicatena_fissicatena | SGB4871_group
Lachnospiraceae | Eisenbergiella_massiliensis | SGB4987
Lachnospiraceae | Blautia_wexlerae | SGB4837_group
Erysipelotrichaceae | Faecalibacillus_intestinalis | SGB6754
Eggerthellaceae | Eggerthella_lenta | SGB14809
Bacteroidales_unclassified | Phocaeicola_dorei | SGB1815
Bacteroidaceae | Bacteroides_uniformis | SGB1836_group
Rikenellaceae | Alistipes_ihumii | SGB2328
Lachnospiraceae | Roseburia_faecis | SGB4925
Lachnospiraceae | Eubacterium_rectale | SGB4933_group
FGB12277 | GGB35068_SGB47850 | SGB47850
FGB42691 | GGB51441_SGB71759 | SGB71759
Eubacteriaceae | Eubacterium_sp_AF15_50 | SGB5042
Clostridiales_unclassified | Clostridiales_unclassified_SGB15145 | SGB15145
Ruminococcaceae | Ruminococcaceae_bacterium | SGB14899
Ruminococcaceae | Faecalibacterium_SGB15346 | SGB15346
Ruminococcaceae | Agathobaculum_butyriciproducens | SGB14993_group
Odoribacteraceae | Odoribacter_splanchnicus | SGB1790
Lachnospiraceae | Fusicatenibacter_saccharivorans | SGB4874
Lachnospiraceae | Dorea_longicatena | SGB4582_group
Lachnospiraceae | Blautia_obeum | SGB4809
Clostridiaceae | Clostridiaceae_bacterium | SGB4269
Clostridiaceae | Clostridiaceae_unclassified_SGB4769 | SGB4769
Tannerellaceae | Parabacteroides_distasonis | SGB1934
Eubacteriaceae | Eubacteriaceae_bacterium | SGB5043
Clostridiaceae | Clostridium_fessum | SGB4705
Christensenellaceae | Christensenellaceae_bacterium | SGB14127
Bacteroidaceae | Bacteroides_clarus | SGB1832
Slope (direction)
1.0
0.5
0.0
−0.5
−1.0
Increasing over visits
Decreasing over visits
Average difference between patients with PFS ≥12 with and without colitis
5.0
2.5
0.0
−2.5
Higher abundance in with colitis
Higher abundance without colitis
0 1 2 3
Visit

Figure S2. Patients with PFS≥12 with and without colitis. Panel (A) shows, for each microbial SGB listed, its slopes in patients with PFS≥12 months with and without colitis, respectively. Red and blue colors indicate whether the focal SGB is increasing or decreasing in its abundance over study visits, respectively. It then shows the average difference between patients with PFS≥12 months with and without colitis across the different study visits. Non-gray cells in the heatmap correspond to the focal SGB's log-fold change in abundance between patients with PFS≥12 months with and without colitis, respectively. Teal cells correspond to study visits for which the abundance of the focal SGB is higher in patients with PFS≥12 months with colitis than in patients with PFS≥12 months without colitis, and vice versa for brown cells (at 90% BCL). Gray cells denote differences between patients with PFS≥12 months with and without colitis whose 90% CI overlapped with zero.

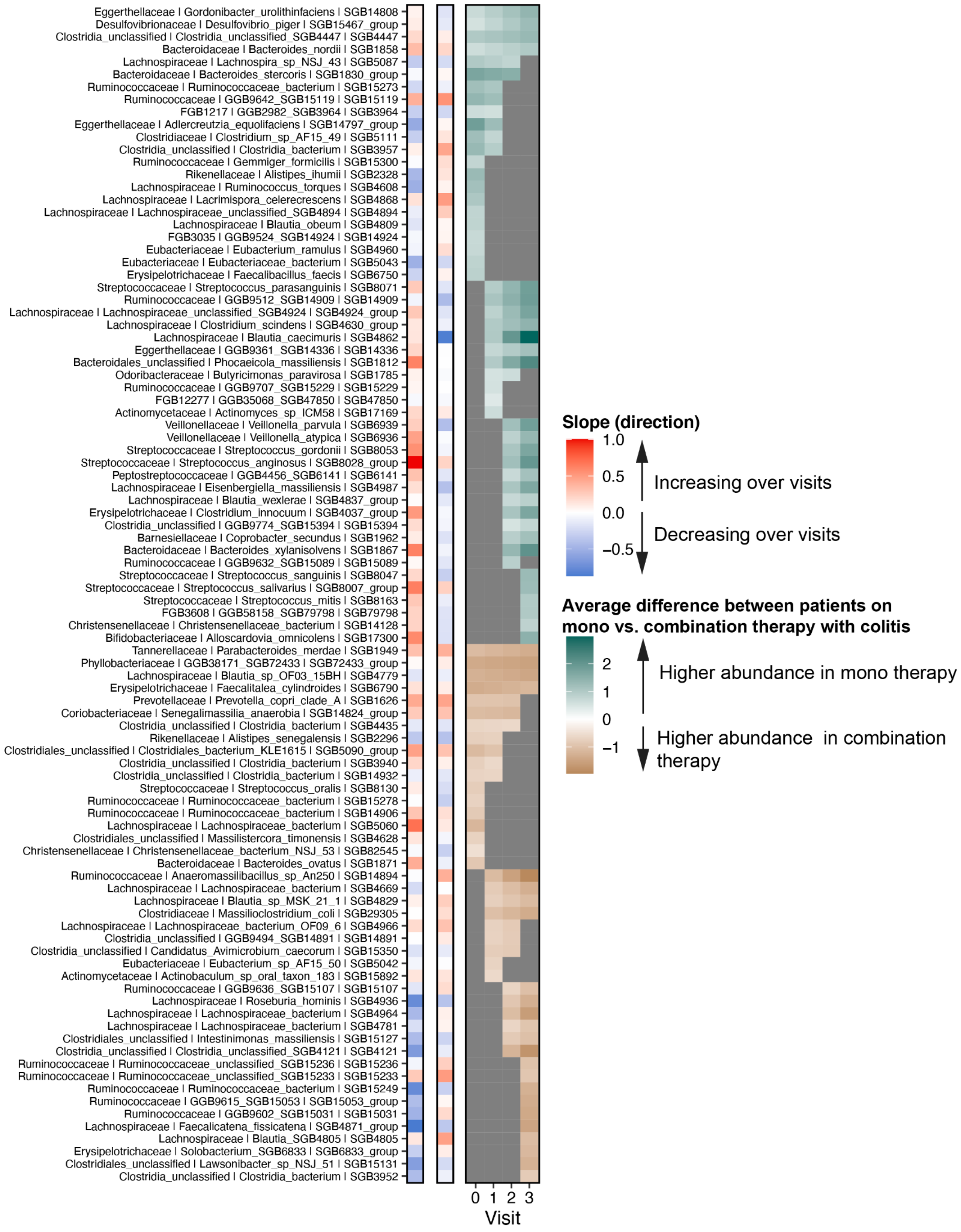

Eggerthellaceae | Gordonibacter_urolithinfaciens | SGB14808
Desulfovibrionaceae | Desulfovibrio_piger | SGB15467_group
Clostridia_unclassified | Clostridia_unclassified_SGB4447 | SGB4447
Bacteroidaceae | Bacteroides_nordii | SGB1858
Lachnospiraceae | Lachnospira_sp_NSJ_43 | SGB5087
Bacteroidaceae | Bacteroides_stercoris | SGB1830_group
Ruminococcaceae | Ruminococcaceae_bacterium | SGB15273
Ruminococcaceae | GGB9642_SGB15119 | SGB15119
FGB1217 | GGB2982_SGB3964 | SGB3964
Eggerthellaceae | Adlercreutzia_equolifaciens | SGB14797_group
Clostridiaceae | Clostridium_sp_AF15_49 | SGB5111
Clostridia_unclassified | Clostridia_bacterium | SGB3957
Ruminococcaceae | Gemmiger_formicilis | SGB15300
Rikenellaceae | Alistipes_ihumii | SGB2328
Lachnospiraceae | Ruminococcus_torques | SGB4608
Lachnospiraceae | Lacrimispora_celerecrescens | SGB4868
Lachnospiraceae | Lachnospiraceae_unclassified_SGB4894 | SGB4894
Lachnospiraceae | Blautia_obeum | SGB4809
FGB3035 | GGB9524_SGB14924 | SGB14924
Eubacteriaceae | Eubacterium_ramulus | SGB4960
Eubacteriaceae | Eubacteriaceae_bacterium | SGB5043
Erysipelotrichaceae | Faecalibacillus_faecis | SGB6750
Streptococcaceae | Streptococcus_parasanguinis | SGB8071
Ruminococcaceae | GGB9512_SGB14909 | SGB14909
Lachnospiraceae | Lachnospiraceae_unclassified_SGB4924 | SGB4924_group
Lachnospiraceae | Clostridium_scindens | SGB4630_group
Lachnospiraceae | Blautia_caecimuris | SGB4862
Eggerthellaceae | GGB9361_SGB14336 | SGB14336
Bacteroidales_unclassified | Phocaeicola_massiliensis | SGB1812
Odoribacteraceae | Butyricimonas_paravirosa | SGB1785
Ruminococcaceae | GGB9707_SGB15229 | SGB15229
FGB12277 | GGB35068_SGB47850 | SGB47850
Actinomycetaceae | Actinomyces_sp_ICM58 | SGB17169
Veillonellaceae | Veillonella_parvula | SGB6939
Veillonellaceae | Veillonella_atypica | SGB6936
Streptococcaceae | Streptococcus_gordonii | SGB8053
Streptococcaceae | Streptococcus_anginosus | SGB8028_group
Peptostreptococcaceae | GGB4456_SGB6141 | SGB6141
Lachnospiraceae | Eisenbergiella_massiliensis | SGB4987
Lachnospiraceae | Blautia_wexlerae | SGB4837_group
Erysipelotrichaceae | Clostridium_innocuum | SGB4037_group
Clostridia_unclassified | GGB9774_SGB15394 | SGB15394
Barnesiellaceae | Coprobacter_secundus | SGB1962
Bacteroidaceae | Bacteroides_xylanisolvens | SGB1867
Ruminococcaceae | GGB9632_SGB15089 | SGB15089
Streptococcaceae | Streptococcus_sanguinis | SGB8047
Streptococcaceae | Streptococcus_salivarius | SGB8007_group
Streptococcaceae | Streptococcus_mitis | SGB8163
FGB3608 | GGB58158_SGB79798 | SGB79798
Christensenellaceae | Christensenellaceae_bacterium | SGB14128
Bifidobacteriaceae | Alloscardovia_omnicolens | SGB17300
Tannerellaceae | Parabacteroides_merdae | SGB1949
Phyllobacteriaceae | GGB38171_SGB72433 | SGB72433_group
Lachnospiraceae | Blautia_sp_OF03_15BH | SGB4779
Erysipelotrichaceae | Faecalitalea_cylindroides | SGB6790
Prevotellaceae | Prevotella_copri_clade_A | SGB1626
Coriobacteriaceae | Senegalimassilia_anaerobia | SGB14824_group
Clostridia_unclassified | Clostridia_bacterium | SGB4435
Rikenellaceae | Alistipes_senegalensis | SGB2296
Clostridiales_unclassified | Clostridiales_bacterium_KLE1615 | SGB5090_group
Clostridia_unclassified | Clostridia_bacterium | SGB3940
Clostridia_unclassified | Clostridia_bacterium | SGB14932
Streptococcaceae | Streptococcus_oralis | SGB8130
Ruminococcaceae | Ruminococcaceae_bacterium | SGB15278
Ruminococcaceae | Ruminococcaceae_bacterium | SGB14906
Lachnospiraceae | Lachnospiraceae_bacterium | SGB5060
Clostridiales_unclassified | Massilistercora_timonensis | SGB4628
Christensenellaceae | Christensenellaceae_bacterium_NSJ_53 | SGB82545
Bacteroidaceae | Bacteroides_ovatus | SGB1871
Ruminococcaceae | Anaeromassilibacillus_sp_An250 | SGB14894
Lachnospiraceae | Lachnospiraceae_bacterium | SGB4669
Lachnospiraceae | Blautia_sp_MSK_21_1 | SGB4829
Clostridiaceae | Massilioclostridium_coli | SGB29305
Lachnospiraceae | Lachnospiraceae_bacterium_OF09_6 | SGB4966
Clostridia_unclassified | GGB9494_SGB14891 | SGB14891
Clostridia_unclassified | Candidatus_Avimicrobium_caecorum | SGB15350
Eubacteriaceae | Eubacterium_sp_AF15_50 | SGB5042
Actinomycetaceae | Actinobaculum_sp_oral_taxon_183 | SGB15892
Ruminococcaceae | GGB9636_SGB15107 | SGB15107
Lachnospiraceae | Roseburia_hominis | SGB4936
Lachnospiraceae | Lachnospiraceae_bacterium | SGB4964
Lachnospiraceae | Lachnospiraceae_bacterium | SGB4781
Clostridiales_unclassified | Intestinimonas_massiliensis | SGB15127
Clostridia_unclassified | Clostridia_unclassified_SGB4121 | SGB4121
Ruminococcaceae | Ruminococcaceae_unclassified_SGB15236 | SGB15236
Ruminococcaceae | Ruminococcaceae_unclassified_SGB15233 | SGB15233
Ruminococcaceae | Ruminococcaceae_bacterium | SGB15249
Ruminococcaceae | GGB9615_SGB15053 | SGB15053_group
Ruminococcaceae | GGB9602_SGB15031 | SGB15031
Lachnospiraceae | Faecalicatena_fissicatena | SGB4871_group
Lachnospiraceae | Blautia_SGB4805 | SGB4805
Erysipelotrichaceae | Solobacterium_SGB6833 | SGB6833_group
Clostridiales_unclassified | Lawsonibacter_sp_NSJ_51 | SGB15131
Clostridia_unclassified | Clostridia_bacterium | SGB3952
0
1
2
3
Visit
Slope (direction)
1.0
0.5
0.0
−0.5
Increasing over visits
Decreasing over visits
Average difference between patients on mono vs. combination therapy with colitis
2
1
0
−1
Higher abundance in mono therapy
Higher abundance in combination therapy

Figure S3. Patients on combination therapy and monotherapy who developed colitis. The figure shows, for each microbial SGB listed, its slopes in patients who developed colitis on combination therapy and monotherapy, respectively. Red and blue colors indicate whether the focal SGB is increasing or decreasing in its abundance over study visits, respectively. It then shows the average difference between patients on combination therapy and monotherapy across the different study visits. Non-gray cells in the heatmap correspond to the focal SGB's log-fold change in abundance between patients on combination and monotherapy, respectively. Teal cells correspond to study visits for which the abundance of the focal SGB is higher in patients who developed colitis on combination therapy compared to those who developed colitis on monotherapy, and vice versa for brown cells (at 90% BCL). Gray cells denote differences between patients with and without colitis whose 90% CI overlapped with zero.

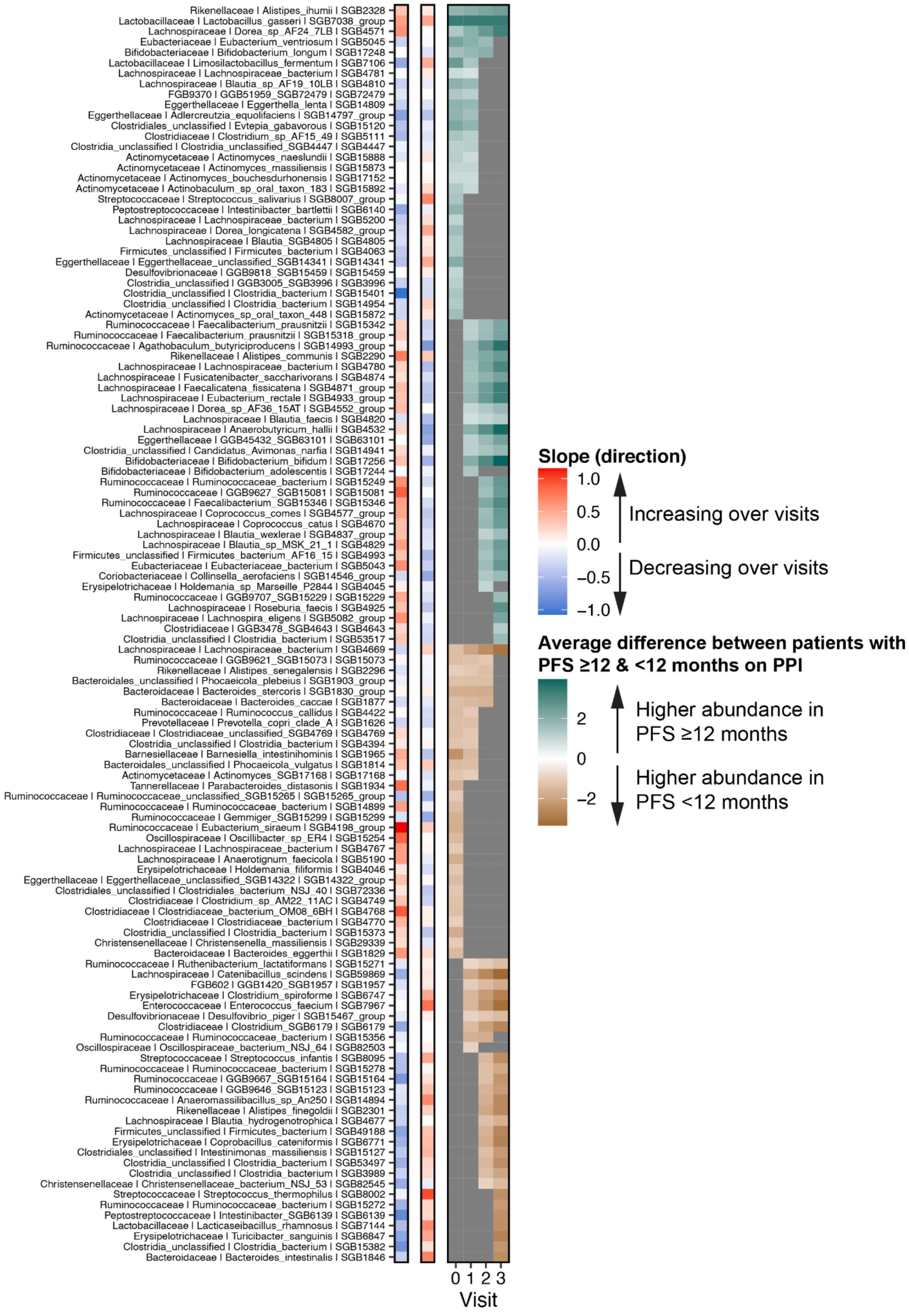
Rikenellaceae | Alistipes_ihumii | SGB2328
Lactobacillaceae | Lactobacillus_gasseri | SGB7038_group
Lachnospiraceae | Dorea_sp_AF24_7LB | SGB4571
Eubacteriaceae | Eubacterium_ventriosum | SGB5045
Bifidobacteriaceae | Bifidobacterium_longum | SGB17248
Lactobacillaceae | Limosilactobacillus_fermentum | SGB7106
Lachnospiraceae | Lachnospiraceae_bacterium | SGB4781
Lachnospiraceae | Blautia_sp_AF19_10LB | SGB4810
FGB9370 | GGB51959_SGB72479 | SGB72479
Eggerthellaceae | Eggerthella_lenta | SGB14809
Eggerthellaceae | Adlercreutzia_equolifaciens | SGB14797_group
Clostridiales_unclassified | Evtepia_gabavorous | SGB15120
Clostridiaceae | Clostridium_sp_AF15_49 | SGB5111
Clostridia_unclassified | Clostridia_unclassified_SGB4447 | SGB4447
Actinomycetaceae | Actinomyces_naeslundii | SGB15888
Actinomycetaceae | Actinomyces_massiliensis | SGB15873
Actinomycetaceae | Actinomyces_bouchesdurhonensis | SGB17152
Actinomycetaceae | Actinobaculum_sp_oral_taxon_183 | SGB15892
Streptococcaceae | Streptococcus_salivarius | SGB8007_group
Peptostreptococcaceae | Intestinibacter_bartlettii | SGB6140
Lachnospiraceae | Lachnospiraceae_bacterium | SGB5200
Lachnospiraceae | Dorea_longicatena | SGB4582_group
Lachnospiraceae | Blautia_SGB4805 | SGB4805
Firmicutes_unclassified | Firmicutes_bacterium | SGB4063
Eggerthellaceae | Eggerthellaceae_unclassified_SGB14341 | SGB14341
Desulfovibrionaceae | GGB9818_SGB15459 | SGB15459
Clostridia_unclassified | GGB3005_SGB3996 | SGB3996
Clostridia_unclassified | Clostridia_bacterium | SGB15401
Clostridia_unclassified | Clostridia_bacterium | SGB14954
Actinomycetaceae | Actinomyces_sp_oral_taxon_448 | SGB15872
Ruminococcaceae | Faecalibacterium_prausnitzii | SGB15342
Ruminococcaceae | Faecalibacterium_prausnitzii | SGB15318_group
Ruminococcaceae | Agathobaculum_butyriciproducens | SGB14993_group
Rikenellaceae | Alistipes_communis | SGB2290
Lachnospiraceae | Lachnospiraceae_bacterium | SGB4780
Lachnospiraceae | Fusicatenibacter_saccharivorans | SGB4874
Lachnospiraceae | Faecalicatena_fissicatena | SGB4871_group
Lachnospiraceae | Eubacterium_rectale | SGB4933_group
Lachnospiraceae | Dorea_sp_AF36_15AT | SGB4552_group
Lachnospiraceae | Blautia_faecis | SGB4820
Lachnospiraceae | Anaerobutyricum_hallii | SGB4532
Eggerthellaceae | GGB45432_SGB63101 | SGB63101
Clostridia_unclassified | Candidatus_Avimonas_narfia | SGB14941
Bifidobacteriaceae | Bifidobacterium_bifidum | SGB17256
Bifidobacteriaceae | Bifidobacterium_adolescentis | SGB17244
Ruminococcaceae | Ruminococcaceae_bacterium | SGB15249
Ruminococcaceae | GGB9627_SGB15081 | SGB15081
Ruminococcaceae | Faecalibacterium_SGB15346 | SGB15346
Lachnospiraceae | Coprococcus_comes | SGB4577_group
Lachnospiraceae | Coprococcus_catus | SGB4670
Lachnospiraceae | Blautia_wexlerae | SGB4837_group
Lachnospiraceae | Blautia_sp_MSK_21_1 | SGB4829
Firmicutes_unclassified | Firmicutes_bacterium_AF16_15 | SGB4993
Eubacteriaceae | Eubacteriaceae_bacterium | SGB5043
Coriobacteriaceae | Collinsella_aerofaciens | SGB14546_group
Erysipelotrichaceae | Holdemania_sp_Marseille_P2844 | SGB4045
Ruminococcaceae | GGB9707_SGB15229 | SGB15229
Lachnospiraceae | Roseburia_faecis | SGB4925
Lachnospiraceae | Lachnospira_eligens | SGB5082_group
Clostridiaceae | GGB3478_SGB4643 | SGB4643
Clostridia_unclassified | Clostridia_bacterium | SGB53517
Lachnospiraceae | Lachnospiraceae_bacterium | SGB4669
Ruminococcaceae | GGB9621_SGB15073 | SGB15073
Rikenellaceae | Alistipes_senegalensis | SGB2296
Bacteroidales_unclassified | Phocaeicola_plebeius | SGB1903_group
Bacteroidaceae | Bacteroides_stercoris | SGB1830_group
Bacteroidaceae | Bacteroides_caccae | SGB1877
Ruminococcaceae | Ruminococcus_callidus | SGB4422
Prevotellaceae | Prevotella_copri_clade_A | SGB1626
Clostridiaceae | Clostridiaceae_unclassified | SGB4769
Clostridia_unclassified | Clostridia_bacterium | SGB4394
Barnesiellaceae | Barnesiella_intestinihominis | SGB1965
Bacteroidales_unclassified | Phocaeicola_vulgatus | SGB1814
Actinomycetaceae | Actinomyces_SGB17168 | SGB17168
Tannerellaceae | Parabacteroides_distasonis | SGB1934
Ruminococcaceae | Ruminococcaceae_unclassified_SGB15265 | SGB15265_group
Ruminococcaceae | Ruminococcaceae_bacterium | SGB14899
Ruminococcaceae | Gemmiger_SGB15299 | SGB15299
Ruminococcaceae | Eubacterium_siraeum | SGB4198_group
Oscillospiraceae | Oscillibacter_sp_ER4 | SGB15254
Lachnospiraceae | Lachnospiraceae_bacterium | SGB4767
Lachnospiraceae | Anaerotignum_faecicola | SGB5190
Erysipelotrichaceae | Holdemania_filiformis | SGB4046
Eggerthellaceae | Eggerthellaceae_unclassified_SGB14322 | SGB14322_group
Clostridiales_unclassified | Clostridiales_bacterium_NSJ_40 | SGB72336
Clostridiaceae | Clostridium_sp_AM22_11AC | SGB4749
Clostridiaceae | Clostridiaceae_bacterium_OM08_6BH | SGB4768
Clostridiaceae | Clostridiaceae_bacterium | SGB4770
Clostridia_unclassified | Clostridia_bacterium | SGB15373
Christensenellaceae | Christensenella_massiliensis | SGB29339
Bacteroidaceae | Bacteroides_eggerthii | SGB1829
Ruminococcaceae | Ruthenibacterium_lactatiformans | SGB15271
Lachnospiraceae | Catenibacillus_scindens | SGB59869
FGB602 | GGB1420_SGB1957 | SGB1957
Erysipelotrichaceae | Clostridium_spiroforme | SGB6747
Enterococcaceae | Enterococcus_faecium | SGB7967
Desulfovibrionaceae | Desulfovibrio_piger | SGB15467_group
Clostridiaceae | Clostridium_SGB6179 | SGB6179
Ruminococcaceae | Ruminococcaceae_bacterium | SGB15356
Oscillospiraceae | Oscillospiraceae_bacterium_NSJ_64 | SGB82503
Streptococcaceae | Streptococcus_infantis | SGB8095
Ruminococcaceae | Ruminococcaceae_bacterium | SGB15278
Ruminococcaceae | GGB9667_SGB15164 | SGB15164
Ruminococcaceae | GGB9646_SGB15123 | SGB15123
Ruminococcaceae | Anaeromassilibacillus_sp_An250 | SGB14894
Rikenellaceae | Alistipes_finegoldii | SGB2301
Lachnospiraceae | Blautia_hydrogenotrophica | SGB4677
Firmicutes_unclassified | Firmicutes_bacterium | SGB49188
Erysipelotrichaceae | Coprobacillus_cateniformis | SGB6771
Clostridiales_unclassified | Intestinimonas_massiliensis | SGB15127
Clostridia_unclassified | Clostridia_bacterium | SGB53497
Clostridia_unclassified | Clostridia_bacterium | SGB3989
Christensenellaceae | Christensenellaceae_bacterium_NSJ_53 | SGB82545
Streptococcaceae | Streptococcus_thermophilus | SGB8002
Ruminococcaceae | Ruminococcaceae_bacterium | SGB15272
Peptostreptococcaceae | Intestinibacter_SGB6139 | SGB6139
Lactobacillaceae | Lacticaseibacillus_rhamnosus | SGB7144
Erysipelotrichaceae | Turicibacter_sanguinis | SGB6847
Clostridia_unclassified | Clostridia_bacterium | SGB15382
Bacteroidaceae | Bacteroides_intestinalis | SGB1846
0 1 2 3
Visit
Slope (direction)
1.0
0.5
0.0
−0.5
−1.0
Increasing over visits
Decreasing over visits
Average difference between patients with PFS ≥12 & <12 months on PPI
2
0
−2
Higher abundance in PFS ≥12 months
Higher abundance in PFS <12 months

Figure S4. **Patients with PFS≥12 and PFS<12 months who used PPIs**. The figure shows, for each microbial SGB listed, its slopes in patients with PFS≥12 and PFS<12 months who used PPIs, respectively. Red and blue colors indicate whether the focal SGB is increasing or decreasing in its abundance over study visits, respectively. It then shows the average difference between patients with PFS≥12 and PFS<12 months across the different study visits. Non-gray cells in the heatmap correspond to the focal SGB's log-fold change in abundance between patients with PFS≥12 and PFS<12 months, respectively. Teal cells correspond to study visits for which the abundance of the focal SGB is higher in patients with PFS≥12 than with PFS<12 months, and vice versa for brown cells (at 90% BCL). Gray cells denote differences between patients who used and did not use PPIs whose 90% CI overlapped with zero.

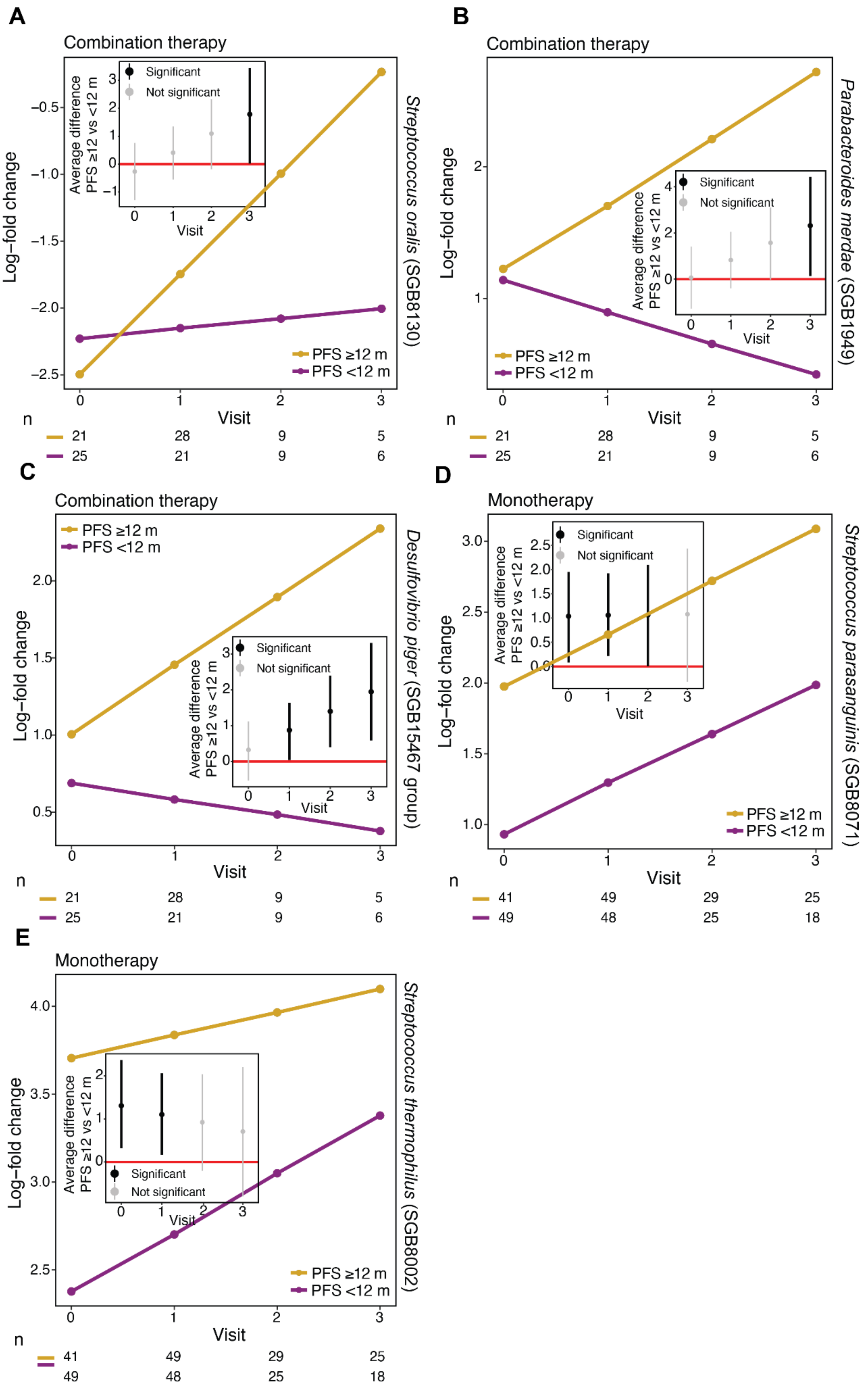
A
Combination therapy
Log−fold change
Visit
n
21 28 9 5
25 21 9 6
PFS ≥12 m
PFS <12 m
Average difference PFS ≥12 vs <12 m
Significant
Not significant
Streptococcus oralis (SGB8130)
B
Combination therapy
Log−fold change
Visit
n
21 28 9 5
25 21 9 6
PFS ≥12 m
PFS <12 m
Average difference PFS ≥12 vs <12 m
Significant
Not significant
Parabacteroides merdae (SGB1949)
C
Combination therapy
Log−fold change
Visit
n
21 28 9 5
25 21 9 6
PFS ≥12 m
PFS <12 m
Average difference PFS ≥12 vs <12 m
Significant
Not significant
Desulfovibrio piger (SGB15467 group)
D
Monotherapy
Log−fold change
Visit
n
41 49 29 25
49 48 25 18
PFS ≥12 m
PFS <12 m
Average difference PFS ≥12 vs <12 m
Significant
Not significant
Streptococcus parasanguinis (SGB8071)
E
Monotherapy
Log−fold change
Visit
n
41 49 29 25
49 48 25 18
PFS ≥12 m
PFS <12 m
Average difference PFS ≥12 vs <12 m
Significant
Not significant
Streptococcus thermophilus (SGB8002)

Figure S5. Pro-inflammatory SGBs increase in patients with PFS≥12 months. Panels (A-E) show five different “dynamical scenarios” exemplified by different “pro-inflammatory” SGBs increasing in patients with PFS≥12 months during ICB. The teal and brown lines correspond to regression slopes across the study visits for patients with PFS≥12 and PFS<12 months, respectively. Panels (A-C) display SGBs under combination therapy, while panels (D-E) show SGBs under monotherapy. The y-axis shows the clr log-ratio value for each study visit (x-axis). The corresponding inset plots show the average difference between patients with PFS≥12 and PFS<12 months at each study visit, including the 90% credible interval; if this does not cross the red zero line, then we deem the focal SGB differentially abundant in the focal study visit.

Supplementary methods

Patients with PFS≥12 months vs. patients with PFS<12 months

The below reference grid shows all combinations of the levels of W_{1-3} for patients with PFS<12 months ($Z = 0$) and patients with PFS≥12 months ($Z = 1$), respectively. The 5th column shows, for each combination, the non-zero coefficients for PFS<12 (A-H) and PFS≥12 (I-P), respectively.

	W_1	W_2	W_3	Patients with PFS<12 months ($Z = 0$)
A	0	0	0	$\beta_0 + \beta_1$
B	1	0	0	$\beta_0 + \beta_1 + \beta_4 + \beta_5$
C	0	1	0	$\beta_0 + \beta_1 + \beta_8 + \beta_9$
D	0	0	1	$\beta_0 + \beta_1 + \beta_{12} + \beta_{13}$
E	1	1	0	$\beta_0 + \beta_1 + \beta_4 + \beta_5 + \beta_8 + \beta_9$
F	0	1	1	$\beta_0 + \beta_1 + \beta_8 + \beta_9 + \beta_{12} + \beta_{13}$
G	1	0	1	$\beta_0 + \beta_1 + \beta_4 + \beta_5 + \beta_{12} + \beta_{13}$
H	1	1	1	$\beta_0 + \beta_1 + \beta_4 + \beta_5 + \beta_8 + \beta_9 + \beta_{12} + \beta_{13}$
	W_1	W_2	W_3	Patients with PFS≥12 months ($Z = 1$)
I	0	0	0	$\beta_0 + \beta_1 + \beta_2 + \beta_3$
J	1	0	0	$\beta_0 + \beta_1 + \beta_2 + \beta_3 + \beta_4 + \beta_5 + \beta_6 + \beta_7$
K	0	1	0	$\beta_0 + \beta_1 + \beta_2 + \beta_3 + \beta_8 + \beta_9 + \beta_{10} + \beta_{11}$
L	0	0	1	$\beta_0 + \beta_1 + \beta_2 + \beta_3 + \beta_{12} + \beta_{13} + \beta_{14} + \beta_{15}$
M	1	1	0	$\beta_0 + \beta_1 + \beta_2 + \beta_3 + \beta_4 + \beta_5 + \beta_6 + \beta_7 + \beta_8 + \beta_9 + \beta_{10} + \beta_{11}$
N	0	1	1	$\beta_0 + \beta_1 + \beta_2 + \beta_3 + \beta_8 + \beta_9 + \beta_{10} + \beta_{11} + \beta_{12} + \beta_{13} + \beta_{14} + \beta_{15}$
O	1	0	1	$\beta_0 + \beta_1 + \beta_2 + \beta_3 + \beta_4 + \beta_5 + \beta_6 + \beta_7 + \beta_{12} + \beta_{13} + \beta_{14} + \beta_{15}$
P	1	1	1	$\beta_0 + \beta_1 + \beta_2 + \beta_3 + \beta_4 + \beta_5 + \beta_6 + \beta_7 + \beta_8 + \beta_9 + \beta_{10} + \beta_{11} + \beta_{12} + \beta_{13} + \beta_{14} + \beta_{15}$

With this reference grid established, we can use the estimated coefficients to compute the marginal mean for patients with PFS≥12 vs. PFS<12 respectively.

$$\widehat{PFS < 12} = \frac{A + B + C + D + E + F + G + H}{8}$$
$$= \underbrace{\beta_0 + 0.5\big(\beta_4 + \beta_8 + \beta_{12}\big)}_{\text{intercepts}} + \underbrace{\big(\beta_1 + 0.5\big(\beta_5 + \beta_9 + \beta_{13}\big)\big)}_{\text{slopes}}X$$

$$\widehat{PFS \geq 12} = \frac{I + J + K + L + M + N + O + P}{8}$$
$$\underbrace{\beta_0 + \beta_2 + 0.5\Big(\beta_4 + \beta_6 + \beta_8 + \beta_{10} + \beta_{12} + \beta_{14}\Big)}_{\text{intercepts}} + \underbrace{\Big(\beta_1 + \beta_3 + 0.5\Big(\beta_5 + \beta_7 + \beta_9 + \beta_{11} + \beta_{13} + \beta_{15}\Big)\Big)}_{\text{slopes}}X$$

Then, finding the difference between the marginal averages for patients with PFS≥12 vs. PFS<12 months, we simply subtract one from the other

$$\widehat{PFS \geq 12} - \widehat{PFS < 12} = \underbrace{\beta_2 + 0.5\big(\beta_6 + \beta_{10} + \beta_{14}\big)}_{\text{intercepts}} + \underbrace{\big(\beta_3 + 0.5\big(\beta_7 + \beta_{11} + \beta_{15}\big)\big)}_{\text{slopes}}X$$

Colitis vs. no colitis

The below reference grid shows all combinations of the levels of Z , W_1 and W_3 for "no colitis" ($W_2 = 0$) and "colitis" ($W_2 = 1$), respectively. The 5th column shows, for each combination, the non-zero coefficients for "no colitis" (A-H) and "colitis" (I-P), respectively.

	Z	W_1	W_3	No colitis ($W_2 = 0$)
A	0	0	0	$\beta_0 + \beta_1$
B	1	0	0	$\beta_0 + \beta_1 + \beta_2 + \beta_3$
C	0	1	0	$\beta_0 + \beta_1 + \beta_4 + \beta_5$
D	0	0	1	$\beta_0 + \beta_1 + \beta_{12} + \beta_{13}$
E	1	1	0	$\beta_0 + \beta_1 + \beta_2 + \beta_3 + \beta_4 + \beta_5 + \beta_6 + \beta_7$
F	0	1	1	$\beta_0 + \beta_1 + \beta_4 + \beta_5 + \beta_{12} + \beta_{13}$
G	1	0	1	$\beta_0 + \beta_1 + \beta_2 + \beta_3 + \beta_{12} + \beta_{13} + \beta_{14} + \beta_{15}$
H	1	1	1	$\beta_0 + \beta_1 + \beta_2 + \beta_3 + \beta_4 + \beta_5 + \beta_6 + \beta_7 + \beta_{12} + \beta_{13} + \beta_{14} + \beta_{15}$
	Z	W_1	W_3	Colitis ($W_2 = 1$)
I	0	0	0	$\beta_0 + \beta_1 + \beta_8 + \beta_9$
J	1	0	0	$\beta_0 + \beta_1 + \beta_2 + \beta_3 + \beta_8 + \beta_9 + \beta_{10} + \beta_{11}$
K	0	1	0	$\beta_0 + \beta_1 + \beta_4 + \beta_5 + \beta_8 + \beta_9$
L	0	0	1	$\beta_0 + \beta_1 + \beta_8 + \beta_9 + \beta_{12} + \beta_{13}$

M	1	1	0	$\beta_0+\beta_1+\beta_2+\beta_3+\beta_4+\beta_5+\beta_6+\beta_7+\beta_8+\beta_9+\beta_{10}+\beta_{11}$
N	0	1	1	$\beta_0+\beta_1+\beta_4+\beta_5+\beta_8+\beta_9+\beta_{12}+\beta_{13}$
O	1	0	1	$\beta_0+\beta_1+\beta_2+\beta_3+\beta_8+\beta_9+\beta_{10}+\beta_{11}+\beta_{12}+\beta_{13}+\beta_{14}+\beta_{15}$
P	1	1	1	$\beta_0+\beta_1+\beta_2+\beta_3+\beta_4+\beta_5+\beta_6+\beta_7+\beta_8+\beta_9+\beta_{10}+\beta_{11}+\beta_{12}+\beta_{13}+\beta_{14}+\beta_{15}$

$$\widehat{\text{No colitis}} = \frac{A+B+C+D+E+F+G+H}{8}$$

$$= \underbrace{\beta_0+0.5(\beta_2+\beta_4+\beta_{12})+0.25(\beta_6+\beta_{14})}_{\text{intercepts}} + \underbrace{(\beta_1+0.5(\beta_3+\beta_5+\beta_{13})+0.25(\beta_7+\beta_{15}))}_{\text{slopes}}X$$

$$\widehat{\text{Colitis}} = \frac{I+J+K+L+M+N+O+P}{8}$$

$$= \underbrace{\beta_0+\beta_8+0.5(\beta_2+\beta_4+\beta_{10}+\beta_{12})+0.25(\beta_6+\beta_{14})}_{\text{intercepts}} + \underbrace{(\beta_1+\beta_9+0.5(\beta_3+\beta_5+\beta_{11}+\beta_{13})+0.25(\beta_7+\beta_{15}))}_{\text{slopes}}X$$

Then, finding the difference between the marginal averages for patients with and without colitis, we simply subtract one from the other

$$\widehat{\text{Colitis}} - \widehat{\text{No colitis}} = \underbrace{\beta_8+0.5(\beta_{10})}_{\text{intercepts}} + \underbrace{(\beta_9+0.5(\beta_{11}))}_{\text{slopes}}X$$

Patients with PFS≥12 months with and without colitis

The below reference grid shows all combinations of the levels of W_1 and W_3 for patients with PFS≥12 months ($Z=1$) without colitis ($W_2=0$) and patients with PFS≥12 months ($Z=1$) with colitis ($W_2=1$), respectively. The 5th column shows, for each combination, the non-zero coefficients for patients with PFS≥12 months without colitis (A-D) and patients with PFS≥12 months with colitis (E-G), respectively.

	W_1	W_3	Patients with ≥ PFS12 m ($Z=1$) without colitis ($W_2=0$)
A	0	0	$\beta_0+\beta_1+\beta_2+\beta_3$
B	1	0	$\beta_0+\beta_1+\beta_2+\beta_3+\beta_4+\beta_5+\beta_6+\beta_7$
C	0	1	$\beta_0+\beta_1+\beta_2+\beta_3+\beta_{12}+\beta_{13}+\beta_{14}+\beta_{15}$
D	1	1	$\beta_0+\beta_1+\beta_2+\beta_3+\beta_4+\beta_5+\beta_6+\beta_7+\beta_{12}+\beta_{13}+\beta_{14}+\beta_{15}$
	W_1	W_3	Patients with ≥ PFS12 m ($Z=1$) with colitis ($W_2=1$)
E	0	0	$\beta_0+\beta_1+\beta_2+\beta_3+\beta_8+\beta_9+\beta_{10}+\beta_{11}$

F	1	0	$\beta_0 + \beta_1 + \beta_2 + \beta_3 + \beta_4 + \beta_5 + \beta_6 + \beta_7 + \beta_8 + \beta_9 + \beta_{10} + \beta_{11}$
G	0	1	$\beta_0 + \beta_1 + \beta_2 + \beta_3 + \beta_8 + \beta_9 + \beta_{10} + \beta_{11} + \beta_{12} + \beta_{13} + \beta_{14} + \beta_{15}$
H	1	1	$\beta_0 + \beta_1 + \beta_2 + \beta_3 + \beta_4 + \beta_5 + \beta_6 + \beta_7 + \beta_8 + \beta_9 + \beta_{10} + \beta_{11} + \beta_{12} + \beta_{13} + \beta_{14} + \beta_{15}$

$$\widehat{\text{without colitis}} = \frac{A + B + C + D}{4}$$

$$= \underbrace{\beta_0 + \beta_2 + 0.5\big(\beta_4 + \beta_6 + \beta_{12} + \beta_{14}\big)}_{\text{intercepts}} + \underbrace{\big(\beta_1 + \beta_3 + 0.5\big(\beta_5 + \beta_7 + \beta_{13} + \beta_{15}\big)\big)}_{\text{slopes}} X$$

$$\widehat{\text{with colitis}} = \frac{E + F + G + H}{4}$$

$$= \underbrace{\beta_0 + \beta_2 + \beta_8 + \beta_10 + 0.5\big(\beta_4 + \beta_6 + \beta_{12} + \beta_{14}\big)}_{\text{intercepts}} + \underbrace{\big(\beta_1 + \beta_3 + \beta_9 + \beta_{11} + 0.5\big(\beta_5 + \beta_7 + \beta_{13} + \beta_{15}\big)\big)}_{\text{slopes}} X$$

Then, finding the difference between the marginal averages for PFS≥12 months with and without colitis, we simply subtract one from the other

$$\widehat{\text{with colitis}} - \widehat{\text{without colitis}} = \underbrace{\beta_8 + \beta_{10}}_{\text{intercepts}} + \underbrace{\big(\beta_9 + \beta_{11}\big)}_{\text{slopes}} X$$
