# Supplementary material for: Longitudinal gut microbiome changes in immune checkpoint blockade-treated advanced melanoma
Source: Nat Med. 2024 Feb 16;30(3):785–96. doi: 10.1038/s41591-024-02803-3 (PMC10957474; doi:10.1038/s41591-024-02803-3)
Supplement: Supplementary file 2 — Reporting Summary. [file 41591_2024_2803_MOESM2_ESM.pdf]

nature portfolio

Corresponding author(s): Johannes R. Björk & Rinse K. Weersma

Last updated by author(s): Dec 15, 2023

Reporting Summary

Nature Portfolio wishes to improve the reproducibility of the work that we publish. This form provides structure for consistency and transparency in reporting. For further information on Nature Portfolio policies, see our Editorial Policies and the Editorial Policy Checklist.

Statistics

For all statistical analyses, confirm that the following items are present in the figure legend, table legend, main text, or Methods section.

n/a	Confirmed	
☐	☒	The exact sample size (*n*) for each experimental group/condition, given as a discrete number and unit of measurement
☒	☐	A statement on whether measurements were taken from distinct samples or whether the same sample was measured repeatedly
☐	☒	The statistical test(s) used AND whether they are one- or two-sided<br>*Only common tests should be described solely by name; describe more complex techniques in the Methods section.*
☐	☒	A description of all covariates tested
☐	☒	A description of any assumptions or corrections, such as tests of normality and adjustment for multiple comparisons
☐	☒	A full description of the statistical parameters including central tendency (e.g. means) or other basic estimates (e.g. regression coefficient) AND variation (e.g. standard deviation) or associated estimates of uncertainty (e.g. confidence intervals)
☐	☒	For null hypothesis testing, the test statistic (e.g. *F*, *t*, *r*) with confidence intervals, effect sizes, degrees of freedom and *P* value noted<br>*Give P values as exact values whenever suitable.*
☐	☒	For Bayesian analysis, information on the choice of priors and Markov chain Monte Carlo settings
☒	☐	For hierarchical and complex designs, identification of the appropriate level for tests and full reporting of outcomes
☒	☐	Estimates of effect sizes (e.g. Cohen's *d*, Pearson's *r*), indicating how they were calculated

Our web collection on statistics for biologists contains articles on many of the points above.

Software and code

Policy information about availability of computer code

Data collection	Data taken from questionnaires, clinical visits and laboratory data from the majority of centres was collected on paper clinical report forms and entered using comma delimited files and excel spreadsheets. No specialist software was required. Exceptions were Oncolifes and POINTING studies. OncoLifes has used an UMCG-developed application called Utopia for data collection. (https://www.ncbi.nlm.nih.gov/pmc/articles/PMC6857242/)<br>Data within the POINTING cohort has been collected using the OpenClinica clinical trial software. (https://www.openclinica.com/)

Data analysis	Microsoft Excel version 2019<br>MetaPhlAn v.4.0 (https://github.com/biobakery/MetaPhlAn)<br>HUMAnN v.3.0 (https://github.com/biobakery/humann)<br>fido (v.1.0.4) R package<br>phyloseq (v.1.42.0) package<br>tidyverse (v.2.0.0) package<br>purrr (v.1.01) package<br>caret (v.6.0-94) R package<br>pROC (v.1.18.0) R package<br>ggsurvfit (v.0.3.0) R package<br>survival (v.3.5-5) R package<br><br>All code is available in the first author's GitHub page (https://github.com/johannesbjork/Longitudinal-gut-microbiome-changes-in-ICB-treated-advanced-melanoma)

For manuscripts utilizing custom algorithms or software that are central to the research but not yet described in published literature, software must be made available to editors and reviewers. We strongly encourage code deposition in a community repository (e.g. GitHub). See the Nature Portfolio guidelines for submitting code & software for further information.

Data

Policy information about availability of data

All manuscripts must include a data availability statement. This statement should provide the following information, where applicable:

- Accession codes, unique identifiers, or web links for publicly available datasets
- A description of any restrictions on data availability
- For clinical datasets or third party data, please ensure that the statement adheres to our policy

The longitudinally profiled metagenomes have been deposited in the European Nucleotide Archive under accession number PRJEB70966. Baseline samples are already deposited under accession number PRJEB43119. All MetaPhlAn4 and HUMAnN3 profiles will also be available within the latest version of curatedMetagenomicData (https://bioconductor.org/packages/curatedMetagenomicData). All relevant patient data used in this study can be requested by emailing the first author (bjork.johannes@gmail.com). The six previously published studies used for validation are available under accession numbers: PRJNA770295; PRJNA541981; PRJNA762360; PRJNA399742; PRJNA397906; PRJEB22893, and PRJEB22894 (see Extended Data Table 1).

Research involving human participants, their data, or biological material

Policy information about studies with human participants or human data. See also policy information about sex, gender (identity/presentation), and sexual orientation and race, ethnicity and racism.

Reporting on sex and gender	Of the 175 patients, 75 were female and 100 male.
Reporting on race, ethnicity, or other socially relevant groupings	Ethnicity was not assessed
Population characteristics	Cohort characteristics are summarized in Table 1. We recruited 175 patients from five distinct cohorts across the Netherlands, the United Kingdom, and Spain treated with ICB for unresectable stage III and stage IV cutaneous melanoma, as previously described 4–10. One hundred seventeen (67%) patients received single agent treatment with an anti-programmed cell death (PD)-1 antibody (nivolumab or pembrolizumab), while 58 (33%) patients received combination therapy with anti-PD-1 and anti-cytotoxic T-lymphocyte-associated antigen (CTLA)-4 antibody (ipilimumab). The Response Evaluation Criteria in Solid Tumors (RECIST v.1.1) were used to determine tumor-response (Online methods). Clinical endpoints were defined as progression-free survival (PFS) at 12 months (PFS12) and overall survival (OS). PFS was defined as the time from the initial immunotherapy to disease progression or death, comparing patients achieving a PFS of 12 months or longer (PFS≥12) and patients with a PFS of less than 12 months (PFS<12). PFS12 was reached by 83 (47%) participants, and the overall median OS was 34.1 months (min=0.39 months, max=93.4 months; OS; censoring date, March 28, 2023). OS was defined for a subset of patients (n=147 patients) as the time in months from initiation of treatment to occurrence of death from any cause. Patients were followed over a maximum period of 7.3 years (median=4.3 years) after providing the first fecal sample. Fecal samples were collected at baseline and three subsequent treatment visits over a period of 12 weeks, Online methods, Figure S1).
Recruitment	We prospectively recruited 128 patients with advanced melanoma who were treated with ICB between August 2015 and January 2020 in the U.K. (PRIMM-UK, n=54) and the Netherlands (PRIMM-NL, n=74, made up of eligible patients from the COLIPI, POINTING and OncoLifeS studies). PRIMM-UK (NCT03643289) is sponsored by East & North Hertfordshire NHS Trust with ethical approval from King's College London. OncoLifeS (METc number 2010/109), COLIPI (METc number 2012/085, NCT02600143) and POINTING (METc number 2018/350, NCT04193956) have all been approved by the Medical Ethical Committee (in Dutch: Medisch Ethische Toetsingsingscommissie or METc) of the University Medical Center Groningen (UMCG) in the Netherlands. OncoLifeS information is available on the Netherlands Trial Register: NTR: https://www.trialregister.nl/trial/7839. Fecal samples were collected from these patients before initiation of ICB and longitudinally at up to four treatment (study) visits: at baseline and before each subsequent treatment cycle over a period of 12 weeks (Figure S1). The time between two samples was 3 or 4 weeks, depending on the treatment regimen, with ipilimumab/nivolumab combination therapy and pembrolizumab monotherapy administered 3-weekly and nivolumab monotherapy administered 4-weekly.<br><br>Patients who fulfilled the following criteria were eligible for the analysis: (i) histologically or cytologically confirmed non-

resectable advanced (stage III or IV) cutaneous melanoma, (ii) treatment with ICB (nivolumab or pembrolizumab) or a combination of ipilimumab and nivolumab) at the recommended dose as a first-line ICI and (iii) 18 years of age or older and (iv) availability of baseline characteristics presented in Table 1.

Written informed consent was obtained from all patients.

Ethics oversight	King's College London (KCL); Medical Ethical Committee of the University Medical Center Groningen (METc UMCG); Manchester Cancer Research Centre (MCRC) Biobank Ethics and MCRC Biobank Access Committee; Ethical committee of Hospital Clinic of Barcelona.

Note that full information on the approval of the study protocol must also be provided in the manuscript.

Field-specific reporting

Please select the one below that is the best fit for your research. If you are not sure, read the appropriate sections before making your selection.

☒ Life sciences ☐ Behavioural & social sciences ☐ Ecological, evolutionary & environmental sciences

For a reference copy of the document with all sections, see nature.com/documents/nr-reporting-summary-flat.pdf

Life sciences study design

All studies must disclose on these points even when the disclosure is negative.

Sample size	As this is an observational study, no power calculations were performed. From 447 samples from 195 patients, after quality control, SGB prevalence filtering and exclusion of samples with missingness in considered clinical metadata, we retained 408 samples from 175 patients.
Data exclusions	We excluded samples of participants with non-metastasized and resectable Stage III melanoma who received ICB's as adjuvant treatment. Moreover patients who were not immunotherapy-naïve were excluded. These exclusion criteria were established prior to this study. We also excluded patients who had any missingness in any of the considered confounder/predictor variables.
Replication	We replicated part of our results in six independent melanoma cohorts.
Randomization	As this was an observational cohort study randomization was not necessary or appropriate to produce our results and conclusions
Blinding	There was no control or placebo arm therefore blinding was not applicable

Reporting for specific materials, systems and methods

We require information from authors about some types of materials, experimental systems and methods used in many studies. Here, indicate whether each material, system or method listed is relevant to your study. If you are not sure if a list item applies to your research, read the appropriate section before selecting a response.

Materials & experimental systems

n/a	Involved in the study	
☒	☐	Antibodies
☒	☐	Eukaryotic cell lines
☒	☐	Palaeontology and archaeology
☒	☐	Animals and other organisms
☐	☒	Clinical data
☒	☐	Dual use research of concern
☒	☐	Plants

Methods

n/a	Involved in the study	
☒	☐	ChIP-seq
☒	☐	Flow cytometry
☒	☐	MRI-based neuroimaging

Clinical data

Policy information about clinical studies

All manuscripts should comply with the ICMJE guidelines for publication of clinical research and a completed CONSORT checklist must be included with all submissions.

Clinical trial registration	NCT02600143; NCT03643289; NCT04193956; https://www.trialregister.nl/trial/7839; MCRC 07/H1003/161+5 and MCRC 13_RIMA_01; HCB/2015/1032; REC Ref 15/NW/0933.
Study protocol	https://www.clinicaltrials.gov
Data collection	Patients within the PRIMM-cohorts were recruited in parallel, using aligned protocols 4. Additional patients, treated between March 2015 and November 2019, were enrolled from cohorts outside the setting of the PRIMM-study: Leeds (n=19); Barcelona (n=11) and Manchester (n=17). Fecal samples were collected at time points similar to those used in our included prospective studies. Patient

samples within the Manchester cohort were collected with written full-informed patient consent under Manchester Cancer Research Centre Biobank ethics application 07/H1003/161+5 (updated in 18/NW/0092) and approval for the work under Manchester Cancer Research Centre Biobank Access Committee application 13_RIMA_01. Barcelona cohort samples were subjected to the ethical committee of Hospital Clínic of Barcelona approval (registry HCB/2015/1032). Data and samples from Leeds were collected in a study named "Developing a blood test of immunity in illness: a study examining the peripheral blood transcriptome in patients with cancer, autoimmune disease, immunodeficiency or iatrogenic immune suppression" (Research Ethics Committee (REC) reference 15/NW/0933). Informed written consent was obtained for collection of samples and data, sharing anonymized data and working with collaborators whether academic or commercial.

Outcomes

Clinical endpoints were defined as progression-free survival (PFS) at 12 months (PFS12) and overall survival (OS). PFS was defined as the time from the initial immunotherapy to disease progression or death, comparing patients achieving a PFS of 12 months or longer (PFS≥12) and patients with a PFS of less than 12 months (PFS<12). PFS12 was reached by 83 (47%) participants, and the overall median OS was 34.1 months (min=0.39 months, max=93.4 months; OS; censoring date, March 28, 2023). OS was defined for a subset of patients (n=147 patients) as the time in months from initiation of treatment to occurrence of death from any cause. Patients were followed over a maximum period of 7.3 years (median=4.3 years) after providing the first fecal sample. Fecal samples were collected at baseline and three subsequent treatment visits over a period of 12 weeks, Online methods, Figure S1).
